# Texas State Board of Medical Examiners

**Published:** 1912-06

**Authors:** 


### Texas State Board of Medical Examiners.

NOTICE OF EXAMINATION.

FORT WORTH, TEXAS, December 1, 1911.

The regular semi-annual examination of the Texas State Board of Medical Examiners will be held in Austin, House of Representatives, June 25-27, 1912. All applicants should be present at 9 a. m. on the morning of June 25th.

The order of examination can not be varied from in any respect and every applicant who desires to be examined must commence the examination on the morning of Tuesday, June 25th. No person will be examined on June 25th except those who have made proper application on the blank forms furnished by the State Board of Medical Examiners and have paid fee on or before June 10th. The fees will be returned to those unable to appear for examination, but unless applicant is prevented by illness from taking the examination he shall forfeit the sum of $2.00 of the fee paid to defray the expenses incurred in arranging for his examination. Applicants are requested to provide themselves with paper 8½ by 11, and envelopes 4½ by 9½ inches in size, in which to enclose each paper when finished. The applicant's fee must be sent to the Secretary at Fort Worth. The applicant must present his diploma for inspection at the examination, not sending it to the Secretary's office. The filing of an application or the taking of an examination does not entitle applicant to practice. The only legal authority being a certificate from the State Board of Medical Examiners, recorded in the district clerk's office of the county of residence.

